# The epithelial polarity axis controls the resting membrane potential and Cl^−^ co-transport in breast glandular structures

**DOI:** 10.1242/jcs.260924

**Published:** 2023-11-09

**Authors:** Albert K. Urazaev, Lei Wang, Yunfeng Bai, Hibret A. Adissu, Sophie A. Lelièvre

**Affiliations:** ^1^ Department of Biological Sciences, Purdue University, West Lafayette, IN 47907, USA; ^2^ School of Liberal Arts, Sciences and Education, Ivy Tech Community College, Lafayette, IN 47905, USA; ^3^ Department of Basic Medical Sciences, Purdue University, West Lafayette, IN 47907, USA; ^4^ Purdue Institute for Cancer Research, West Lafayette, IN 47907, USA; ^5^ Relation Gene-Environment-REGEN Unit, Institut de Cancérologie de l'Ouest (ICO), Angers 49055, France

**Keywords:** 3D cell culture, Gap junction, Apical polarity, Basal polarity, Breast cancer, Cell nucleus

## Abstract

The membrane potential (MP) controls cell homeostasis by directing molecule transport and gene expression. How the MP is set upon epithelial differentiation is unknown. Given that tissue architecture also controls homeostasis, we investigated the relationship between basoapical polarity and resting MP in three-dimensional culture of the HMT-3522 breast cancer progression. A microelectrode technique to measure MP and input resistance reveals that the MP is raised by gap junction intercellular communication (GJIC), which directs tight-junction mediated apical polarity, and is decreased by the Na^+^/K^+^/2Cl^−^ (NKCC, encoded by *SLC12A1* and *SLC12A2*) co-transporter, active in multicellular structures displaying basal polarity. In the tumor counterpart, the MP is reduced. Cancer cells display diminished GJIC and do not respond to furosemide, implying loss of NKCC activity. Induced differentiation of cancer cells into basally polarized multicellular structures restores widespread GJIC and NKCC responses, but these structures display the lowest MP. The absence of apical polarity, necessary for cancer onset, in the non-neoplastic epithelium is also associated with the lowest MP under active Cl^−^ transport. We propose that the loss of apical polarity in the breast epithelium destabilizes cellular homeostasis in part by lowering the MP.

## Teaser

**Summary:** There is a central role for epithelial polarity in controlling cell homeostasis via an impact on the membrane potential.

## INTRODUCTION

All cells possess a resting membrane potential (MP) that determines how they respond to external stimulation and controls transmembrane transport. Typically, the high MP observed in excitable cells, like muscle cells and neurons (−45 to −90 mV) is established by concentration gradients of ions across the semipermeable cell membrane. In cells considered ‘non to poorly excitable’ the lower range MP (−9 to −40 mV) is cell type specific, suggesting that the MP level might correspond to specialized tissue functions. Indeed, an increase in MP is necessary for stem cell differentiation (Bhavsar et al., 2019) and a decrease in MP leads to the downregulation of differentiation markers (Sundelacruz et al., 2013). In an epithelium, the MP sets the transepithelial charge for transport through the cell layer. The MP also regulates morphological features of cells via an impact on actin organization (Chifflet and Hernández, 2012). Notably, hyperpolarization influences actin compaction along adherens junctions, which increases junction stability (Nin et al., 2009). This control over tissue architecture (i.e. the organization of cells, their internal components and their extracellular matrix), itself known to direct cellular functions (Lelièvre and Chittiboyina, 2018; Soleas et al., 2020), reinforces the importance of the MP in differentiation.

Three decades ago, the depolarization of cancer cell membranes was recorded in a breast biopsy (Marino et al., 1994). Computer modeling confirmed the lower electric field strength in tumors compared to normal tissues due to an altered MP that could affect functions throughout the cell, even at the nucleus level (Sree et al., 2011). Not surprisingly, MP depolarization was proposed to participate in the ‘dedifferentiated’ status of cancer and the activation of cancerous transformation (Lobikin et al., 2012; Yang and Brackenbury, 2013). Nevertheless, the cellular features that set MP levels in the rigorous architectural design of an epithelium remain to be identified.

Through dedifferentiation of non-neoplastic epithelial cells and redifferentiation of their derived cancer cells in three-dimensional (3D) cell culture, we show that the MP is influenced by elements of the polarity axis, integrating hemidesmosomes and gap junctions, and that the sole absence of apical polarity (as shown by the altered distribution of tight junctions) leads to an MP lower than in cancer cells due to sustained Cl^−^ transport. Here, we have unveiled a central role for epithelial polarity in controlling cell homeostasis via an impact on the MP.

## RESULTS

### The resting membrane potential differs between phenotypically normal epithelial structures and tumor nodules

**Fig. 1.**
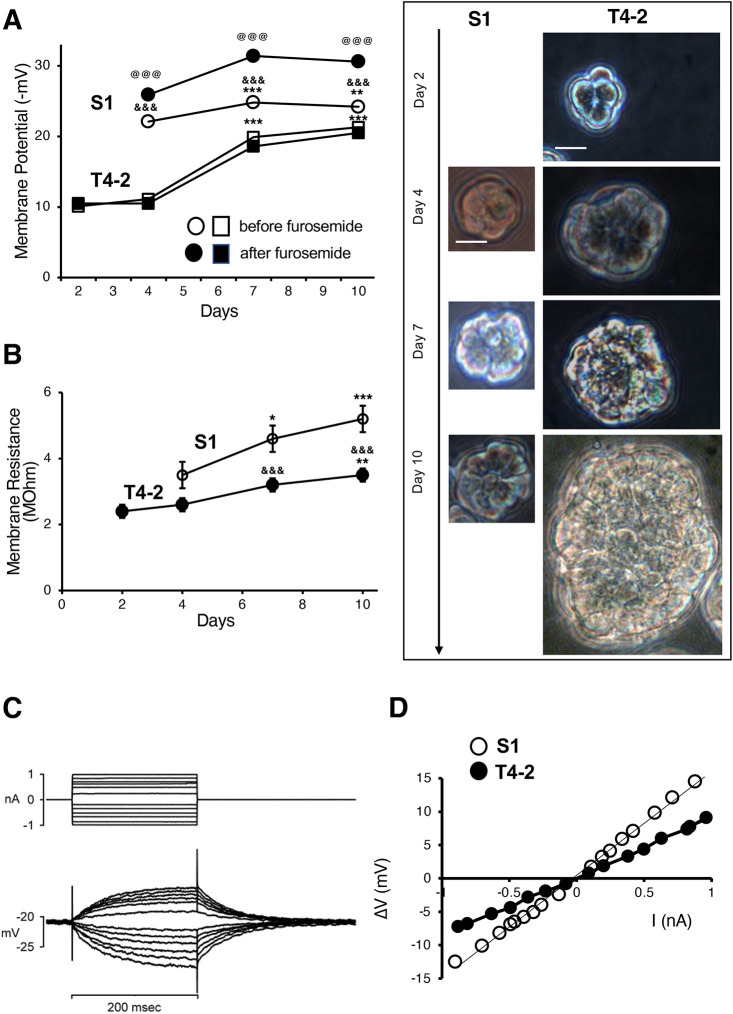
**MP and NKCC co-transport activity vary depending on cell phenotypes.** Non-neoplastic S1 cells and cancerous T4-2 cells were cultured in 3D for 10 days. (A) Mean±s.e.m. MP in S1 cells (circles) and T4-2 cells (squares) measured at days 2, 4 ,7 and 10 before (white symbols) and after (black symbols) treatment with 10^−4^ mol/l furosemide for 5 min. Repeated-measures ANOVA revealed significant group [*F*(61143)=332.42, *P*<0.001] and trial effects [*F*(11053)=109.5, *P*<0.001] and significant group×trial interaction [*F*(61053)=17.65, *P*<0.001; shown here]. Right panel: brightfield images of multicellular structures. For MP measurement *n*=3 cell culture vessels per condition; 50 cells analyzed per vessel, so 150 cells per condition (see Table S1 for MP measurement numbers). Notes: (1) day 2 MP for S1 cells would correspond to single cells; (2) the s.e.m. range (0.4–0.6 mV) makes the error bars indistinguishable from the chart symbols at this scale. (B) *R*_in_ measurements following a hyperpolarizing pulse (−1 nA, 200 ms) for S1 (white circles) and T4-2 (black circles) cells. The repeated-measures ANOVA revealed a significant group effect [*F*(1370)=156.3; *P*<0.001], significant trial effect [*F*(3830)=218.0; *P*<0.001] and significant group×trial interaction [*F*(3830)=34.0; *P*<0.001; shown here]. Results are mean±s.e.m. (*n*=40–84; see Table S1 for Rin measurement numbers). Representative images of S1 and T4-2 multicellular structures in 3D culture are shown on the right of A and B. The acinus picture for day 7 in the S1 panel is part of a series of images corresponding to an experiment with injection of Lucifer Yellow that is shown Fig. 4D. Scale bars: 20 µm. (C) 200 ms pulses with various amplitudes ranging from −1 nA (hyperpolarizing) to +1 nA (depolarizing) were used to record the current–voltage relationship of the membrane potential of cultured cells (top graph). Each current injection triggered a corresponding change of MP (Δ*V*), which was recorded (lower graph). Similarly, a single 200 ms/1 nA square hyperpolarizing pulse was used to monitor the input resistance of individual cells. The input resistance was calculated using Ohm's law, by dividing the change in MP, Δ*V* by the amplitude of injected current (−1 nA). Representative of 172 repeats. (D) Representative recordings (of 172 repeats), made in S1 cells (open circles) and T4-2 cells (filled circles) after 4 days in culture, showing that the current–voltage relationship of the membrane potential is linear. *P<0.05; **P<0.01; ***^,&&&,@@@^*P*<0.001 (least squares means post hoc test of interaction) where asterisks (*) are time course comparison to day 4 in S1 cells and day 2 in T4-2 cells; ampersands (&) are comparison between S1 and T4-2 cells at each time point; and at (@) symbols are comparison of the effect of furosemide at each time point.

The non-neoplastic HMT 3522 S1 breast epithelial cells are a reliable model for full glandular differentiation, which includes the formation of a basoapical polarity axis in the presence of basement membrane components, in 3D cell culture (Plachot et al., 2009). Using a standard microelectrode technique, we measured the resting MP over the 10-day morphogenesis process (starting from one cell to produce glandular structures or acini with 20–30 cells; Plachot et al., 2009). The average MP significantly rose by 2.7 mV between day 4 and 7 starting from −22.1±0.5 mV (mean±s.e.m.) and thus, ending with a number that is more negative (or higher, or hyperpolarized) compared to the reference point in the medium. There was no further change at day 10 (Fig. 1A; Table S1). For the small number of single cells present at days 4 and 7, the MP had similar values of −10.2±0.4 mV (*n*=46) and −10.2±0.7 mV (*n*=13; *P*=0.83) that were significantly different (*P*<0.001) from those measured in the cells forming acini in the same dishes. Thus, the rise in MP measured during glandular morphogenesis was a result of the normal proliferation and differentiation of the cells and not a simple consequence of the time spent in culture. Some cells exhibited rapid low-amplitude MP oscillations during recordings (Fig. S1A), which is consistent with results from murine mammary epithelial cells in culture and might be related to responses to hormones and growth factors (e.g. insulin, EGF, prolactin and cortisol) (Enomoto et al., 1986).

To verify the positive link between glandular differentiation and MP level, we used the S1-derived triple negative breast cancer T4-2 cells, which proliferate actively and form tumors devoid of polarity in 3D culture (Chandramouly et al., 2007). As expected, the average MP in these cells was consistently lower compared to the S1 cells, especially on day 4 (−11.1 mV; Fig. 1A; Table S1); although it rose at day 7 of culture, it remained ∼5 mV lower (-19.9 mV versus −24.8 mV) than the MP in differentiated S1 cells. We did not detect MP oscillations in T4-2 cells (Fig. S1B).

The cell membrane acts as a resistor that hinders the passive flow (current, *I*) of ions. Therefore, we investigated the membrane input resistance (*R*_in_) to further understand the observed differences in MP. Cells were injected for 200 ms with a hyperpolarizing pulse of 1 nA amplitude. For the constant current that we deliver in these experiments, the increase of charge measured on the cell membrane (Δ*V*) is dependent on its *R*_in_, as predicted by Ohm's law [Δ*V*=*I*×*R*_in_]. We first confirmed that the difference in potential (Δ*V*) across the cell membrane was linearly proportional to the current through it by injecting varying depolarizing and hyperpolarizing currents with the amplitudes in diapason from +1 nA to −1 nA (Fig. 1C,D). When measured in the developing acini the average *R*_in_ progressively increased from 3.5±0.4 MΩ (day 4), to 4.6±0.3 MΩ (day 7) and 5.2±0.4 MΩ (day 10) (mean±s.e.m.; Fig. 1B; Table S1), consistent with the rise in MP. The *R*_in_ in T4-2 cells slowly rose compared to the initial 2.3±0.3 MΩ (day 2) to reach 3.5±0.2 MΩ in 10-day tumors, hence being always lower than the *R*_in_ in S1 cells at corresponding days (Fig. 1B; Table S1). Thus, changes in MP might be linked to modifications in the number and/or activity of ion channels and transporters in the cell membrane.

### Active Cl^−^ transport controls the MP level in basally polarized multicellular structures

The size of T4-2 cells is about twice that of S1 cells (Chandramouly et al., 2007), which might account, at least in part, for the lower input resistance and, thus, lower MP that we measured in these cancer cells (Berman et al., 1994). Cell size is controlled by furosemide-sensitive active Na^+^/K^+^/2Cl^−^ co-transporter (NKCC; encoded by *SLC12A1* and *SLC12A2*), which influences the MP and is present in many types of epithelial cells (Haas and Forbush, 2000), as it is the case in the S1 cells (Fig. S2). By loading the cells with Cl^−^ ions, active internally directed NKCC transport would affect the MP through the shift in equilibrium Cl^−^ potential (Ecl) to a more positive level compared to the equilibrium potential for K^+^ ions (Ek). Indeed, when the S1 cells were exposed to 0.1 mM furosemide, their MP was hyperpolarized by 3.8 mV on day 4 and by ∼6.5 mV on days 7–10 of 3D culture, suggesting that NKCC is functional in these cells and maintains the elevated internal concentration of Cl^−^ (Fig. 1A; Table S1). In contrast, furosemide did not affect the MP of T4-2 cells (Fig. 1A; Table S1), indicating that Cl^−^ is passively distributed across the cell membrane and it is at equilibrium with the MP. Thus, NKCC is either absent or inactive in these cells. Importantly, the repeated-measures ANOVA of *R*_in_ measurements did not reveal a significant group×trial interaction [*F*(6506)=1.23; *P*=0.29] in furosemide-treated cells, suggesting that the inhibition of Cl^−^ transport did not affect the ion conductance. We conclude that furosemide-induced increase of MP in S1 cells is likely due to a direct effect on active Cl^−^ transport. Moreover, ANOVA revealed a significant group effect [*F*(6595)=9.82; *P*<0.0001], supporting our conclusion that the *R*_in_ in S1 cells is different from the *R*_in_ in T4-2 cells.

**Fig. 2.**
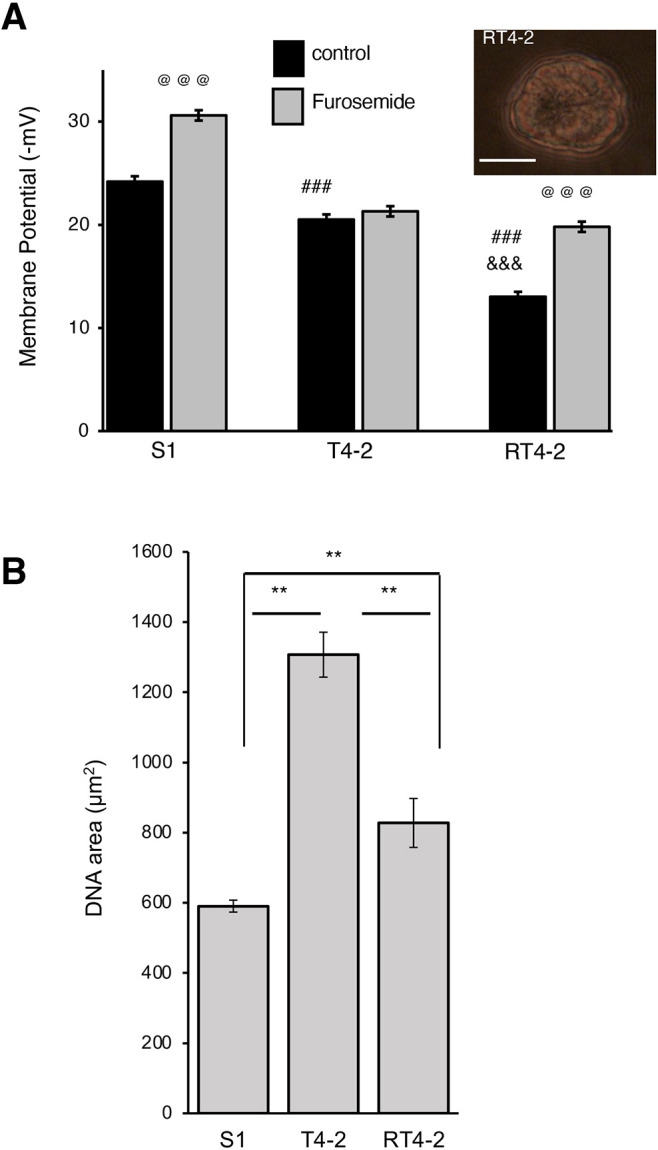
**Treatment of cancer cells to acquire basal polarity restores NKCC activity and influences nuclear morphometry.** Non-neoplastic S1 cells, cancerous T4-2 cells untreated or treated with 100 nM AG1478 to promote partial phenotypic reversion (RT4-2) were cultured in 3D for 10 days. (A) Mean±s.e.m. MP at day 10 of culture before and after treatment with 10^−4^ mol/l furosemide for 5 min. Inset: brightfield image of a multicellular structure formed by RT4-2 cells. Scale bar: 20 µm. Repeated-measures ANOVA revealed significant group [*F*(2565)=250.57; *P*<0.001] and trial effects [*F*(1557)=172.31; *P*<0.001] and significant group×trial interaction [*F*(2557)=30.471; *P*<0.001]. For MP measurement *n*=3 cell culture vessels per condition; 50 cells analyzed per vessel, so 150 cells per condition. (B) Mean±s.e.m. DNA staining area at day 10 of 3D culture measured with ImageJ (*n*=3 cell culture replicates; >29 nuclei analyzed). **P<0.01; ^###,&&&,@@@^*P*<0.001 [least squares means posthoc test of interaction (A); unpaired two-tailed *t*-test (B)]. For A, at (@) symbols effect of furosemide; hash (#) symbols for comparison to control S1 cells; ampersands (&) for comparison between T4-2 and RT4-2 cells in control. For B, comparisons are between S1 and T4-2, S1 and RT4-2, and T4-2 and RT4-2.

Blocking specific signaling pathways induces the differentiation of T4-2 cancer cells into basally polarized, growth-arrested multicellular structures devoid of apical polarity and lumen (called the ‘reverted RT4-2 phenotype’) (Chandramouly et al., 2007; Weaver et al., 1997). To confirm the importance of glandular morphogenesis in setting MP levels, we treated T4-2 cells from day 1 of 3D culture with 100 nM of AG1478, an inhibitor of the EGFR pathway well-established as a means to induce a reverted phenotype in these cells (Chandramouly et al., 2007; Wang et al., 1998; Weaver et al., 1997). Surprisingly, the average MP in RT4-2 cells was lower than in both S1 and T4-2 cells (Fig. 2A; Table S1). The size and cell number of multicellular structures formed by RT4-2 cells were comparable to S1 acini (Weaver et al., 1997), which suggests that the drop in MP might not be related to a change in cell size. Furosemide hyperpolarized the RT4-2 cells by 6.8 mV, bringing the MP to the level observed in T4-2 cells after 10 days in 3D culture, which shows that the glandular-like differentiation of cancer cells is associated with reactivation of Cl^−^ co-transport. Yet, the significantly different area of the bulk of DNA as measured by morphometric analysis of DAPI staining, which is routinely used in cancer pathology to evaluate phenotypical drift (Hamilton et al., 2014), confirms that the glandular-like differentiation of RT4-2 cells corresponds to a different situation compared to the glandular differentiation of the non-neoplastic cells (Fig. 2B).

**Fig. 3.**
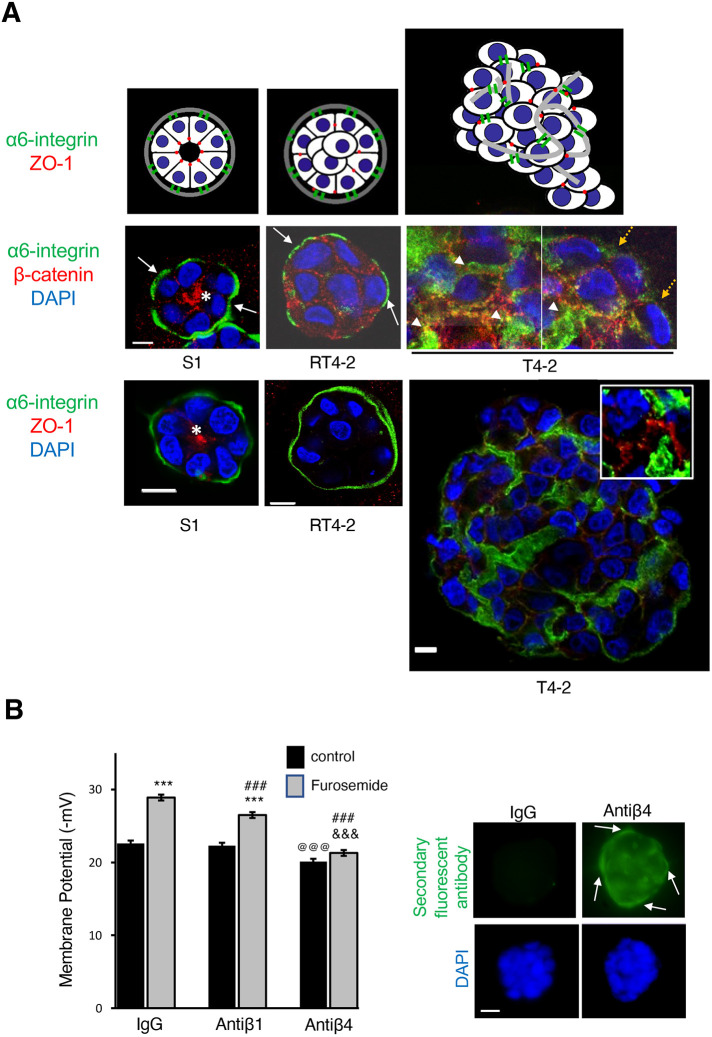
**Inhibiting basal polarity cancels NKCC co-transport activity.** (A) Representative fluorescence images for more than three repeats of α6-integrin (basal polarity marker; green), β-catenin [which is concentrated centrally in the presence of apical tight junctions (Bazzoun et al., 2019); red] and ZO-1 (tight junction marker; red) from 10-day multicellular structures formed by S1, T4-2 and RT4-2 cells in 3D cultures. Indicated in the middle panel are the presence (white plain arrows) and absence (orange dashed arrows) of basally located α6-integrin at the structure periphery, the accumulation of α6-integrin in-between cells (arrowheads) and the apical location of β-catenin (white asterisks). In the lower panel, the inset shows magnified ZO-1 and α6-integrin staining. Nuclei are stained with DAPI (blue). Note: the images with dual staining for α6-integrin and ZO-1 belong to batches of cells different from those used for dual α6-integrin and β-catenin staining. For T4-2, two adjacent portions of a tumor are shown. The cartoons display the multicellular organization, with the absence of a lumen in RT4-2 and T4-2 nodules. (B) S1 cells were cultured in 3D for 10 days in the presence of non-specific immunoglobulins (IgG), and β1- (Antiβ1) or β4-integrin (Antiβ4) function-blocking antibody. Shown is the mean±s.e.m. MP before and after treatment with 10^−4^ mol/l furosemide for 5 min. Arrows indicate the presence of anti-β4 integrin antibody at the periphery of the multicellular structure detected by immunostaining only with a secondary antibody (green), given that the primary antibodies were introduced initially in live-cell cultures. In contrast, staining with secondary antibody only confirms the absence of non-specific binding of negative control IgG. Nuclei are stained with DAPI (blue). For MP measurement *n*=3 cell culture vessels per condition; 50 cells analyzed per vessel, so 150 cells per condition. Repeated-measures ANOVA revealed significant group [*F*(2447)=61.8; *P*<0.0001] and trial effects [*F*(1447)=132.7; *P*<0.001] and significant group×trial interaction [*F*(2447)=19.9; *P*<0.001]. ***^,@@@,###,&&&^*P*<0.001 (least squares means post hoc test for the group×trial interaction) where asterisks (*) are for the effect of furosemide, at (@) symbols are for the difference compared to IgG or control, hash (#) symbols are for the difference compared to IgG plus furosemide, and ampersand (&) symbols are for the difference between anti-β1-integrin plus furosemide and anti-β4-integrin plus furosemide. Scale bars: 5 µm (A, middle panels); 10 µm (A, lower panels; B).

Epithelial differentiation is established and maintained by the basoapical polarity axis. In RT4-2 multicellular structures, only basal polarity linked to hemidesmosomes is present; apical polarity linked to tight junctions is absent and there is no lumen (Chandramouly et al., 2007) (Fig. 3A). Incubation of S1 cells with function-blocking antibodies against β4-integrin to disrupt the basoapical polarity axis (Weaver et al., 2002; Vidi et al., 2012a,b) significantly decreased the MP at day 10 of 3D culture compared to controls [immunoglobulins (IgG) and function-blocking antibodies against β1-integrin]. Long-term incubation with anti-β4-integrin does not significantly alter cell survival (Fig. S3), ruling out the possibility of MP depolarization being associated with apoptosis (Franco et al., 2006). Importantly, the exposure of anti-β4-integrin-treated cells to furosemide did not significantly change their MP (Fig. 3B; Table S1), confirming a lack of NKCC activity in cells missing basal polarity. Moreover, furosemide significantly hyperpolarized cells exposed to IgG or anti-β1-integrin antibodies by 6.3 and 4.2 mV, respectively (Fig. 3B; Table S1), which is similar to its action in plain S1 cells (Fig. 1A). These latter results were expected given that blocking β1-integrin signaling does not interfere with polarity formation (Weaver et al., 1997), and the Cl^−^ transport was predicted to remain active under these circumstances. Thus, basal polarity is necessary to maintain the activity of the Cl^−^ co-transporter.

### Blocking intercellular gap junction communication influences MP but not Cl^−^ transport

**Fig. 4.**
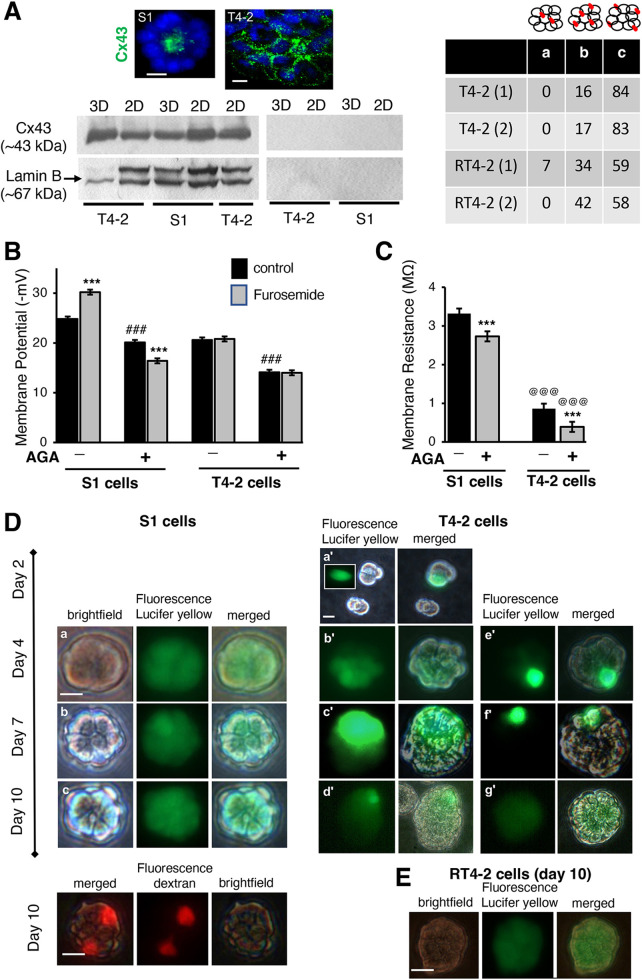
**Blocking gap junctions reduces the MP but does not inhibit NKCC co-transport.** Non-neoplastic S1 cells, cancerous T4-2 cells untreated or treated with 100 nM AG1478 to promote partial phenotypic reversion (RT4-2) were cultured in 2D (on plastic) (A) or in 3D for 10 (A,D,E) or 12 (B,C) days. (A) Representative images of more than three repeats for Cx43 staining (green) in a differentiated acinus, with apical localization (S1 cells), and in a portion of a tumor, with a dispersed and heterogeneous localization (T4-2 cells). Left, representative western blot for Cx43 with lamin B used as loading control, with non-specific binding control (only secondary antibody used) to the right of this panel; there is no significant difference in Cx43 levels between S1 and T4-2 cells (*P*=0.3161; unpaired two-tailed *t*-test, InStat GraphPad, *n*=3 biological replicates); right: number of multicellular structures with Cx43 (shown in red on the drawings), (a) mostly at the center of the structure, (b) throughout the structure, (c) throughout the structure without concentration at the center (*n*=2 biological replicates). (B) Mean±s.e.m. MP before and after treatment with 10^−4^ mol/l furosemide (5 min) of S1 and T4-2 cells previously exposed (+) or not (−) to AGA (50 µM; days 8 to 12 of 3D culture). Repeated-measures ANOVA revealed a significant group effect [*F*(3810)=296.3; *P*<0.001], no significant trial effect [*F*(1624)=1.83; *P*=0.18] and significant group×trial interaction (*F*(3624)=37.1; *P*<0.001). For MP measurement *n*=3 cell culture vessels per condition; 50 cells analyzed per vessel, so 150 cells per condition (see Table S1 for MP and *R*_in_ measurement numbers). (C) Same conditions as in B, with measurement of membrane resistance. Repeated-measures ANOVA revealed significant group effect [*F*(3410)=114.62; *P*<0.001; shown here], significant trial effect [*F*(1226)=6.96; *P*<0.01] and no significant group×trial interaction [*F*(3226)=2.48; *P*=0.062]. Results are mean±s.e.m. (*n*=41–118). ***^,@@@,###^*P*<0.001 (least squares means post hoc test for the group×trial interaction) where asterisks (*) are for effect of furosemide (B) or effect of AGA treatment (C), hash symbols (#) are for the difference between AGA-treated and nontreated S1 or T4-2 cells, and at symbols (@) are for the difference between S1 or T4-2 cells (both control and AGA-treated). (D,E) Representative fluorescence and corresponding brightfield images of multicellular structures injected during a 10-day culture period with Lucifer Yellow (green) in one cell followed by 2–10 min incubation to allow the dye to spread through gap junctions. All structures from S1 [5/5 d4, 5/5 d7, 9/9 d10; (a–c)] and RT4-2 (3/3, d10) cells showed total diffusion; T4-2 structures showed no (a′,d′,e′,f′; 3/3 d2, 4/6 d4, 3/8 d7, 2/6 d10), partial (b′,c′; 1/6 d4, 5/8 d7, 2/6 d10) diffusion, and rarely (g′; 1/6 d4, 2/6 d10) total diffusion. These ratios are the number of structures corresponding to the named (total, partial or no) diffusion pattern over the total number of structures observed at the different culture time points in days (d). The integrity of the membranes after penetration with the microelectrode is exemplified by two cells injected with fluorescent dextran (red) in a glandular structure (bottom, left). Scale bars: 10 µm.

To identify the architectural features that raise the MP in acini formed by S1 cells, although they have active Cl^−^ co-transport (normally setting the MP at lower level), we investigated the role of gap junctions. We have shown previously that connexin 43 (Cx43; also known as GJA1)-mediated gap junctions locate lateroapically in polarized acini and control apical polarity (Bazzoun et al., 2019). Here, we are reporting that this connexin is present in T4-2 and RT4-2 structures, but it is not distributed homogeneously (Fig. 4A). Incubation of 3D cultures with 50 µM of 18-α-glycyrrhetinic acid (AGA), a standard approach to functionally block gap junctions, from days 8 to 12, led to MPs in S1 and T4-2 cells that were, respectively, 4.7 and 6.5 mV lower than in untreated S1 and T4-2 cells (Fig. 4B; Table S1). As expected, furosemide exposure of AGA-treated T4-2 cells did not change their MP; however, it further decreased the MP of AGA-treated S1 cells (Fig. 4B; Table S1). The measurement of *R*_in_ in these experiments revealed a significant group effect [*F*(3410)=114.62, *P*<0.0001, Fig. 4C; Table S1], indicating that AGA treatment decreased the membrane resistance in both S1 and T4-2 cells by presumably impairing electrical connections among cells. The absence of a significant group×trial interaction [*F*(3226)=2.48, *P*=0.062] established that furosemide did not change membrane resistance in both S1 and T4-2 cells treated with AGA. We confirmed that S1 and T4-2 cells in 3D culture displayed functional gap junctions, although to a greatly lesser extent in T4-2 cells, via microinjection of Lucifer Yellow in one cell of several multicellular structures (Fig. 4D). Therefore, functional gap junctions control the level of MP, but blocking gap junctions does not stop Cl^−^ transport. Noticeably, RT4-2 glandular structures also possess functional gap junctions (Fig. 4E), indicating that the higher MP in acini of non-neoplastic S1 cells is not due to these communication channels.

### The absence of apical polarity is associated with a decrease in MP in the glandular epithelium

**Fig. 5.**
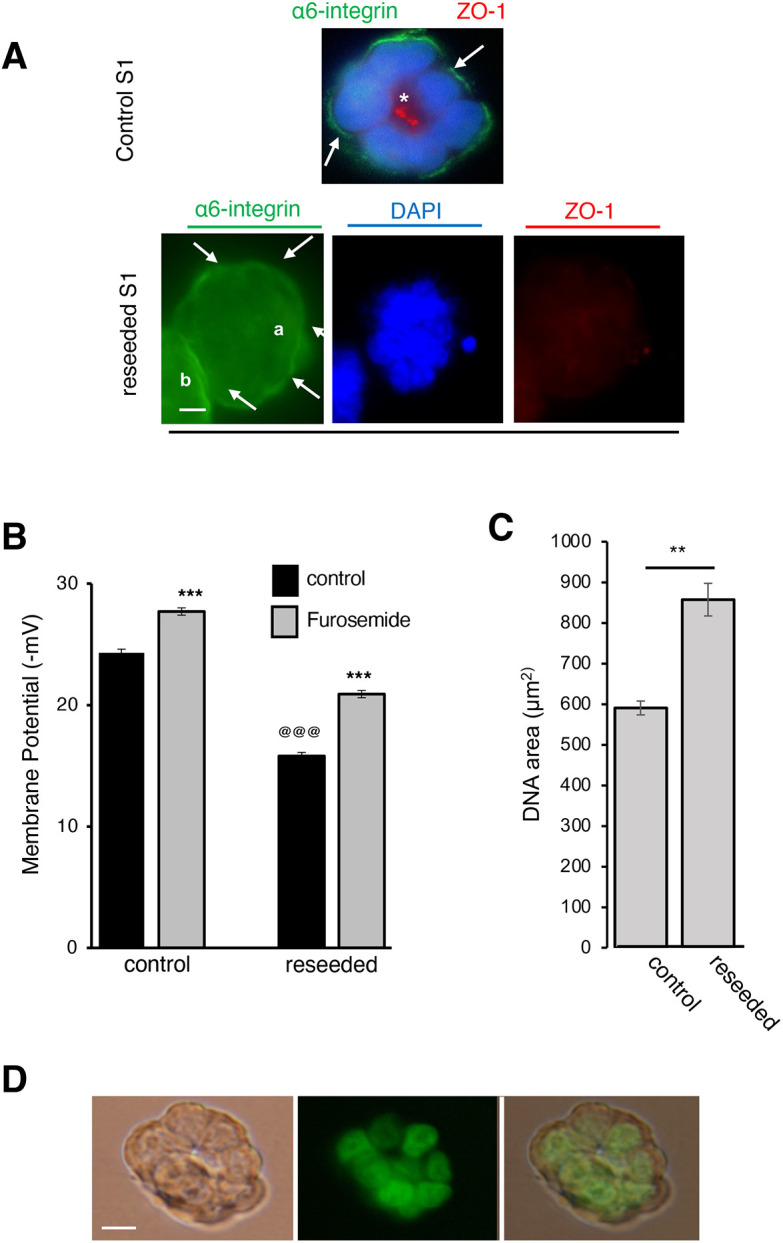
**The lack of apical polarity in S1 structures without inhibition of NKCC co-transport is associated with low MP.** Non-neoplastic S1 cells were cultured in 3D (Matrigel drip) for 8 days to induce the formation of basoapically polarized glandular structures [control]. S1 cells were also cultured in collagen I for 7 days to induce the formation of multicellular structures without polarity, released from collagen with collagenase and reseeded in the presence of Matrigel for 13.5 h (eighth day) [reseeded], which is sufficient time to restore basal polarity but not apical polarity (Chandramouly et al., 2007). (A) Representative fluorescence images from three repeats of α6-integrin (in green) (arrows) and ZO-1 (in red; the asterisk indicates an apical location) in multicellular structures. Note: a and b mark different glandular structures; ZO-1 is very diffuse in the reseeded structure. (B) Mean±s.e.m. MP before and after treatment with 10^−4^ mol/l furosemide for 5 min. The repeated-measures ANOVA revealed significant group [*F*(1298)=558.5, *P*<0.0001] and trial effects [*F*(1298)=238.62, *P*<0.0001] and significant group×trial interaction [*F*(1298)=9.22, *P*=0.0026; shown here]. For MP measurement *n*= 3 cell culture vessels per condition; 50 cells analyzed per vessel, so 150 cells per condition. ***^,@@@^*P*<0.001 (least squares means posthoc test of interaction) with asterisks (*) indicating effect of furosemide and at symbol (@) difference between control cells and reseeded cells. (C) Mean±s.e.m. nuclear area at day 10 of 3D culture measured with ImageJ. *n*=3 cell culture replicates; 44 nuclei analyzed for control and 33 nuclei for reseeded. **P<0.01 (unpaired two-tailed *t*-test) (D) Representative fluorescence and corresponding brightfield images from three repeats of reseeded multicellular structures injected with Lucifer Yellow (green) in one cell followed by 10 min incubation to allow the dye to spread through gap junctions. Scale bars: 5 µm.

A major difference between multicellular structures formed by non-neoplastic S1 and revertant RT4-2 cells is the lack of apical polarity, as shown by the absence of tight junctions at the lateroapical cell–cell contacts upon differentiation mimicry (Chandramouly et al., 2007). To assess the impact of apical polarity on the MP, the S1 cells were cultured in collagen I for 10 days to block the formation of the polarity axis. The resulting multicellular structures were placed in Matrigel (made of basement membrane components) for ∼12 h. This process of collagen I to Matrigel reseeding restores basal polarity within 12 h, as indicated by the presence of markers of hemidesmosomes (α6β4 integrins), without yet reestablishing apical polarity, as shown by the absence of apical ZO-1 staining (Fig. 5A), hence avoiding the use of chemical treatment to alter apical polarity (Chandramouly et al., 2007). Indeed, basally polarized S1 multicellular structures had a significantly lower MP when compared to fully polarized control cells solely cultured in Matrigel (Fig. 5B; Table S1). The MP level was similar to the one measured in RT4-2 cells (Fig. 2) and did not display oscillations (see Fig. S1B).

The cells in only-basally-polarized structures displayed an average DNA staining area significantly higher than that of fully differentiated S1 cells and not significantly different from RT4-2 cells (*P*=0.71), confirming that cells with a low MP that accompany apical polarity loss have an altered organization all the way to the cell nucleus (Fig. 5C). The S1 cells of reseeded multicellular structures responded to furosemide by 5.1 mV hyperpolarization, validating the reestablishment of basal polarity, as indicated by the activity of Cl^−^ co-transport (Fig. 5B; Table S1). The multicellular structures also kept functional gap junctions (Fig. 5D).

We propose that the presence of apical polarity is what sets the higher level of MP upon full glandular differentiation and that loss of apical polarity, a phenomenon associated with breast cancer risk (Bazzoun et al., 2019; Tenvooren et al., 2019), triggers plasma membrane depolarization.

## DISCUSSION

In poorly excitable tissues, like epithelia, the MP has been suggested to regulate transepithelial flow of water and solutes associated with a secretory activity (Chifflet and Hernández, 2012), and to modulate cell proliferation and differentiation (Yang and Brackenbury, 2013). Here, our results indicate that the MP level is influenced by the polarity axis, an essential architectural feature of the differentiated epithelium, the alteration of which is necessary for cancer onset and progression (Chandramouly et al., 2007; Bazzoun et al., 2019).

We attribute the increase in MP during glandular differentiation to the progressive organization of the basoapical polarity axis. *In vitro*, acinus morphogenesis typically starts from one cell and results from cell division with the establishment of basal polarity, followed by growth arrest and apical polarity formation (Plachot et al., 2009). We show that the presence of basal polarity is associated with NKCC activity, which keeps the cells at a low MP. Indeed, pumping Na^+^, K^+^ and Cl^−^ into the cytoplasm increases the intracellular Cl^−^ concentration ([Cl^−^]_in_; Linzell and Peaker, 1971), thus, moving the Ecl to more positive values, away from the Ek, and depolarizing the membrane. These results are in line with NKCC1 participation in mammary gland morphogenesis shown during murine development (Shillingford et al., 2002). The resulting Cl^−^ accumulation likely serves a glandular function by acting as a driving force for the secretion of ions and water across the apical membrane (Shennan and Peaker, 2000) and into the luminal space that opens into the external environment in exocrine glands. This efflux of ions, organic substances and water through the apical membrane serves as a signal for the membrane transport activation on the basolateral side of the cell to maintain its water and electrolyte content along with its volume. The outward secretion through the apical membrane creates a luminal milieu in which the ionic concentrations are different compared to the interstitial fluid. One such secreted ions is Cl^−^, which exits the cell by diffusion through Cl^−^ channels located only in the apical membrane (Anderson et al., 1992; Kamikawa et al., 2016). Consequently, the activation of the inwardly directed NKCC on the basolateral side of the cell will compensate for the cellular loss of Cl^−^ and replenish its water content. Another consequence of unequal ion concentrations between the lumen and the interstitium and of different ion permeabilities in apical and basolateral membranes (Blatchford and Peaker, 1988), is different MPs in these membranes. This difference creates a transepithelial potential (TEP), which is calculated as apical MP subtracted from basolateral MP. The TEP is relatively small (with the apical side usually negative; Blaug et al., 2003), but it is enough to create a transepithelial electrical force that affects the bulk transport of charged substances across the glandular epithelium, further contributing to functional epithelial organization. Our experimental technique does not allow us to directly evaluate the MP on the apical side of the cell without opening (and therefore destroying) the epithelial structures.

Future molecular investigations of apical MP might provide further insight into the establishment of MP (electrogenesis) in developing normal epithelial tissues. Notably, Cx43-mediated gap junctions influence the establishment of apical polarity (Bazzoun et al., 2019), but Cx43 is not part of tight junction complexes per se. Knocking out zonula occludens proteins (e.g. ZO-1, also known as TJP1) that directly control tight junction formation and, thus, apical polarity, and observing electrogenesis over acinus development in 3D cell culture will be necessary to demonstrate that hallmark structures of apical polarity are involved. Another way is to target differentiated acini with conditional knockout of ZO-1 by genome editing, which should decrease the MP to the lower levels observed in only basally polarized structures (i.e. reseeded S1 and RT4-2 nodules). However, interactions between ZO proteins and Cx43, as well as between ZO-1 and catenins have been reported (see Bazzoun et al., 2019 and references therein), which suggests that additional interference with molecules of tight junction complexes might be necessary to ascertain the participation of tight junctions in the control of MP. Possible targets include PaTJ (Shin et al., 2005), which is involved in the Crumbs complex, and PAR3, which is involved in the PAR complex (Chen and Macara, 2005), but altering these proteins would potentially affect adherens junctions; hence, the timing of disruption of the latter cell–cell contacts compared to tight junctions will be crucial. Getting deeper mechanistic insight requires exploring pathways like the phosphoinositide 3-kinase (PI3K) pathway, which controls tight junction formation, even under the influence of GJIC, in the breast epithelium (Bazzoun et al., 2019). Modulating the PI3K pathway through specific inhibitors in addition to AGA treatment might help distinguish between a GJIC-mediated impact and a tight-junction mediated impact on MP, given that blocking PI3K activation prevents the loss of apical polarity (Tenvooren et al., 2019).

Another potential area of research is to reveal the role of other ion transporters, particularly K^+^ and possibly Na^+^ channels (Blaug et al., 2003) and the Na^+^/K^+^-pump, in the electrogenesis in developing normal epithelium as well as in malignant cellular structures, in which the NKCC is nonfunctional and cannot therefore contribute to lowering their MP. We speculate that a depolarized MP in tumor cells might be a result of decreased activity of the Na^+^/K^+^-ATPase, which would change the intracellular concentrations of Na^+^ and K^+^, and of an altered permeability for these ions in the basolateral membrane.

Whether the significantly different level of MP in T4-2 cancer cells compared to differentiated S1 cells and to only basally differentiated RT4-2 and reseeded S1 cells is commonly due to the absence of polarity should be confirmed with additional cells. If the presence of the sole basal polarity with active NKCC co-transport generally sets the lower levels of MP, these levels should also be observed in ductal carcinoma *in situ* HMT-3522 S2 cells and MCF10AT cells, as well as in MCF10A non-neoplastic cells that do not display apical polarity (Plachot et al., 2009). Moreover, the many available cell lines that represent different forms of non-polarized, invasive breast ductal carcinomas should display MP levels above those of structures with only basal polarity; however, their MP level should be below that of fully polarized structures formed by S1 cells. Finally, reverting cancer phenotypes to form fully polarized epithelial structures would consolidate the role of apical polarity in setting the MP of phenotypically normal differentiation; but to the best of our knowledge, to this date, methods to reestablish apical polarity via induced differentiation of cancer cells have not been reported.

As S1 cells proliferate, differentiate and organize into an epithelial structure (or acinus) from day 4 to day 10 in 3D culture, some of these cells exhibit rapid (80–100 Hz) low amplitude (2–3 mV) oscillations of their MP. Simultaneously, these nonsecreting epithelial structures develop a tiny lumen reaching ∼10 µm in diameter (Plachot et al., 2009). Interestingly, the MP oscillations were not observed in T4-2, RT4-2 or reseeded S1 multicellular structures, all of which lack a lumen (Chandramouly et al., 2007). A separate study might reveal whether these oscillations are linked to lumen formation, with subsequent fluctuations caused by lumen development and expansion.

In this study, we also show that the MP level depends on GJIC. Importantly, the MP level is independent from the cancerous or noncancerous nature of cells, but it is contingent upon the differentiation stage that is reflected all the way to the cell nucleus by changes in DNA staining area (Fig. 2; Fig. 5B,C). Indeed, cancer cells induced to form basally polarized and growth-arrested structures (RT4-2), as well as non-neoplastic cells partially differentiated (reseeded S1 multicellular structures with only basal polarity) display a low MP (−13 versus −15.8 mV) along with NKCC activity and extended GJIC.

The MP reaches its maximum level at day 7 of glandular morphogenesis, corresponding to the final stage of differentiation (Plachot et al., 2009); it is −24.5 mV on average, consistent with the relatively low range observed in epithelia (Enomoto et al., 1986). It is understandable that the architectural differentiation of the acinus influences the MP, given that it is associated with a specific organization of elements known to affect ion channels. Notably, the actin skeleton forms a ring of fibers connected to tight junctions (Fanning et al., 2012), and cytoskeletal actin organization is known to control the polarized expression, intracellular trafficking and activity of Na^+^, Cl^−^ and K^+^ channels (Mazzochi et al., 2006). Moreover, the apicolateral localization of gap junctions (Bazzoun et al., 2019) might foster the electrical coupling of ion channels located apically, like K^+^ and Cl^−^ channels, including cystic fibrosis transmembrane conductance regulator (CFTR) (Mazzochi et al., 2006), which maintain homeostasis. Especially, CFTR promotes strong cell–cell contacts and prevents epithelial to mesenchymal transition (Zhang et al., 2013).

The rise in MP during glandular morphogenesis is validated by the parallel increase in membrane resistance, suggesting developmental alterations in the cell membrane ion conductance. Consequently, there might be changes in the responsiveness of cells to external stimuli as the tissue develops. In addition to soluble factors, it could affect the response to mechanostimuli due to modifications in shape and size, as cells organize into a duct or an acinus with a spheroidal geometry. Indeed, mechanotransduction involves the cytoskeleton, which is itself sensitive to the MP and capable of propagating electrical signals from the membrane to deeper cellular structures (Tuszyński et al., 2004). The coupling of mechanical stress and electrophysiology has been reported with stem cell differentiation, changes in cell shape and mechanically induced changes in gene expression (Cox et al., 2019).

The MP in cancer cells forming tumors is lower than in normal tissue, confirming previous reports (Marino et al., 1994; Sree et al., 2011). The lower MP is not due to NKCC activity as it is unaltered by NKCC inhibitor furosemide (see Fig. 2A). Two possibilities linked to tumor architecture that might lower the MP in cancer cells are (1) limited GJIC (Fig. 4D), as GJIC is normally associated with MP increase (Binggeli and Weinstein, 1986; Fig. 4B); and (2) bigger cell size, as an increase in cell size usually promotes MP depolarization (Berman et al., 1994).

Surprisingly, the MP of glandular-like structures without apical polarity is even lower than in cancer cells (−13 to −15.8 versus −20.5 mV). It is, at least in part, associated with the activity of NKCC, in which Cl^−^ movement is an essential component to establish the MP via the control of [Cl^−^]_in_. The disruption of apical polarity appears necessary for cancer onset (as it controls cell cycle entry) (Chandramouly et al., 2007; Bazzoun et al., 2019) and accompanies risk factors, such as high body mass index (BMI) (Tenvooren et al., 2019). Here, we identify a strong decrease in MP that is associated with sustained Cl^−^ co-transporter activity along with an absence of apical polarity. We postulate that such changes in electrophysiological conditions might be another characteristic of an increased risk for breast cancer (Fig. 5C). Membrane depolarization has been shown to induce cancerous transformation in *Xenopus* embryo (Lobikin et al., 2012). Cl^−^ ions might act as a second messenger by modifying both the expression of various genes (Valdivieso and Santa-Coloma, 2019) and protein phosphorylation in signaling pathways (Piala et al., 2017). We propose that understanding how a strong decrease in MP associated with sustained Cl^−^ co-transporter activity might influence gene transcription involved in cancer onset would be an interesting area of development for future studies.

## MATERIALS AND METHODS

### Cell culture

All 2D and 3D cell culture protocols in serum-free H14 DMEM are published for the HMT-3522 non-neoplastic breast epithelial S1 cells and S1-derived cancer T4-2 cells (Chandramouly et al., 2007). The cell lines were obtained from the laboratory of Dr Mina J. Bissell in 2000; they are now available commercially (under the name of HMT-3522 S1 and HMT-3522 T4-2 cells). We maintain these cells and check for phenotypes according to protocols that we have previously described (Vidi et al., 2012a,b). For 3D cell culture, we used the 5% drip method with Matrigel^TM^ (BD Biosciences) or embedding in collagen I (Cellagen Solution AC-5, ICN biomedicals, Costa Mesa, CA, USA) before release with collagenase and reseeding in Matrigel (Chhetri et al., 2019). To induce reversion towards a normal phenotype, T4-2 cells in Matrigel drip were treated from day 1 with 100 nM AG1478 (Cell Signaling Technology) prepared in DMSO (Chandramouly et al., 2007). In some experiments, acini in Matrigel drip were incubated with 20 µg/ml function blocking antibodies [anti-β4-integrin, MAB2058Z, MilliporeSigma, rat IgG anti-human β1-integrin AIIB2, a kind gift from Caroline Damsky, University of California San Francisco, CA, USA, or nonspecific immunoglobulins (IgG), Jackson Immunoresearch, West Grove, PA, USA] from days 7 to 10 (with renewal of medium containing antibodies on day 9), as previously described (Weaver et al., 2002). For blocking GJIC, cells in Matrigel drip were incubated from days 8 to 12 with 18α-glycerrhitinic acid (AGA; Sigma-Aldrich) prepared in DMSO, as described previously (Bazzoun et al., 2019).

### Electrophysiological and gap junction assessments

The MP was measured between the inside (=negative potential) and the outside (=zero potential) of the basal portion of the basolateral membrane. Borosilicate capillary tubing [1.0 mm outer diameter (OD)×0.75 mm internal diameter (ID)/Fiber; Frederick Haer & Co, Bowdoinham, ME, USA] and a P-97 microelectrode puller (Sutter Instruments; Novato, CA) were used to make standard thin-walled glass microelectrodes (30-30-0, Frederick-Haer & Co) with tip resistance of 15–25 MΩ when filled with 3 M potassium acetate. Membrane potentials and current injections were registered with a high-impedance negative-capacitance AxoClamp 2B amplifier (Axon Instruments, Union City, CA). A 200 ms/1 nA square hyperpolarizing pulse was used for current injections to monitor input resistance in individual cells, and 200 ms pulses with various amplitudes ranging from −1 nA (hyperpolarizing) to +1 nA (depolarizing) were used to record the current–voltage relationship (Fig. 1C). All experiments revealed a linear current-voltage relationship for the MP of S1 cells (days 4–10) and T4-2 cells (days 2-10) (Fig. 1D). A dual-output S88 stimulator (Grass-Telefactor, West Warwick, RI, USA) with SIU5 stimulus isolation units (Grass-Telefactor) enabled intracellular stimulations. Intracellular signals were filtered with a low-pass 4302 Dual 24 dB/octave filter (Ithaco, Ithaca, NY). Data were digitized via a Digidata 1322A interface and collected with Axoscope9 (Axon Instruments).

During recordings, cell cultures were kept at room temperature. Three 35 mm dishes were used in each series of experiments, with 50 cells from different multicellular structures recorded in every dish before and after 5 min incubation in fresh culture medium containing furosemide (10^−4^ mol/l; Sigma-Aldrich). Hence, the MP was measured in 150 cells per condition and per treatment. Injections with pulses of current to record the input resistance and the current–voltage relationship were performed in 5–20 cells per dish.

For assessment of gap junction communication, Lucifer Yellow (2.5%) and Dextran (2%) dissolved in 0.15 M LiCl were microinjected in single cells of multicellular structures as detailed previously (Bazzoun et al., 2019). Hyperpolarizing pulses for ionophoretical injections were 4–5.5 nA/500 ms at a frequency of 0.75 Hz for 1 min. Penetration of the cell membrane was determined by monitoring the MP. Please note that all electrophysiological procedures (MP recording, current or dye injections) were undertaken by penetrating cells on their basolateral side, and the potentials were measured between the inner side of the membrane and the incubation medium (=ground). The transepithelial potential, an electrical difference between the outer sides of basal and apical membranes in normal epithelial layers, was not recorded in our experiments. The measured resting MP has always a negative value (−mV) and a higher MP means a more negative potential.

### Western blotting and immunostaining procedures

We used standard techniques for the preparation of total cell extracts followed by SDS-PAGE with 30 µg proteins, and for immunostaining after permeabilization in cytoskeleton buffer directly in 4-well chambered slides, as described previously (Bazzoun et al., 2019). Primary monoclonal antibodies were against lamin B (60 ng/ml, Ab16048 Abcam, Cambridge, MA), β-catenin (2.5 µg/ml, C19220; BD Biosciences), NKCC1 (5 µg/ml, Ab59791, Abcam), ZO-1 (2.5 µg/ml, 33-9100, Life Technologies; Grand Island, NY, USA) and polyclonal antibodies were against α6-integrin (rat, 5 µg/ml, clone NK1-GoH3, EMD Millipore, Billerica, MA, USA), connexin 43 (rabbit, 0.5 mg/ml; C6219, Sigma-Aldrich). DNA was stained with DAPI. We have used antibodies against lamin B, ZO-1, β-catenin, α6-integrin, and Cx43 previously in several publications (see Bazzoun et al., 2019 for a recent one); the antibody used for NKCC1 immunostaining was also reported in the published work of others (for instance Yan et al., 2018). Examples of Cx43 patterns on western blots are included in Fig. S4.

### Microscopy and nuclear morphometry analysis

Fluorescence images were recorded with an inverted IX70 microscope (Olympus) equipped with a Retiga 1300 camera (QImaging, Surrey, BC, Canada) and 40×, NA 1.4 fluor lens, a laser scanning MRC-1024 UV (Bio-Rad) linked to a Diaphot 300 inverted microscope (Nikon, Tokyo, Japan) and oil immersion 60×, numerical aperture (NA) 1.4 apochromatic and 40×, NA 1.4 fluor lenses, and a multiphoton laser scanning confocal Radiance 2100 MP (Bio-Rad Laboratories, Hemel Hampstead, UK) linked to a TE2000 (Nikon, Tokyo, Japan) inverted microscope, with oil immersion 60×, numerical aperture 1.4 apochromatic lens. Brightfield images were taken either with the IX70 microscope or a Nikon Diaphot 300 equipped with a QImaging color Retiga 1300*i* FAST. Confocal images were processed using Confocal Assistant 4.02 (Bio-Rad Laboratories) and assembled in Adobe Photoshop 6.0 (Adobe Systems, San Jose, CA, USA). This software was also used for adjustment of luminosity and contrast of fluorescence images. DAPI-stained nuclei were recorded and analyzed with ImageJ as previously described (Chittiboyina et al., 2018).

### Statistical analyses

For electrophysiological assessment, repeated-measures analysis of variance (ANOVA) was used for the analysis of differences between independent and dependent variables, with a least squares test for post-hoc analyses of significant main effects and interactions (SAS 9.1.3, SAS Institute, Cary, NC, USA). To analyze differences in MP, we used one-way ANOVA with Tukey–Kramer multiple comparisons post test (InStat 3.06, GraphPad, San Diego, CA, USA). For the analyses of nuclear area and apoptotic nuclei we used an unpaired two-tailed *t*-test for every two groups. The differences were considered significant if *P*≤0.05. Data are expressed as mean±s.e.m.

## Supplementary Material

10.1242/joces.260924_sup1 Supplementary information

Table S1. Original data for results presented. Tab 1 has data for Fig. 1A, tab 2 has data for Fig. 1B, tab 3 has data for Fig 2A, tab 4 has data for Fig 3B, tab 5 has data for Fig. 4B, tab 6 has data for Fig. 4C, and tab 7 has data for Fig 5B.
