# Supplementary material for: The epithelial polarity axis controls the resting membrane potential and Cl− co-transport in breast glandular structures
Source: J Cell Sci. 2023 Nov 9;137(5):jcs260924. doi: 10.1242/jcs.260924 (PMC10651101; doi:10.1242/jcs.260924)
Supplement: Supplementary information [file joces-137-260924-s1.pdf]

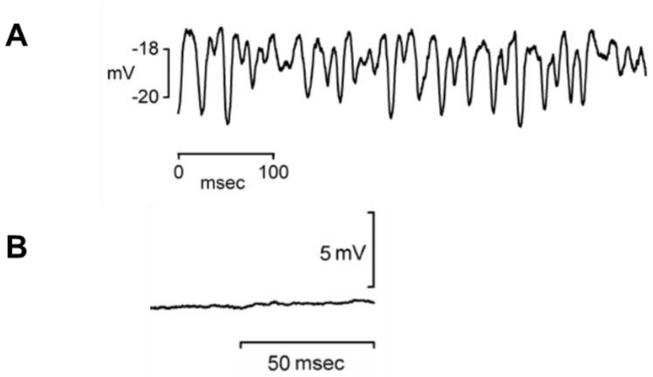

Fig. S1. Particularities of MP and experimental set up. A, a small number of S1 cells exhibited spontaneous fluctuations of their membrane potential in a form of irregular waves with an amplitude of 2-3 mV and a frequency of 80-100 Hz (top graph). Since the membrane potential in such cells is constantly changing, we did not use these cells for statistical analysis of the mean membrane potential. **B**, Such waves of membrane potential were not observed (i.e., there was no oscillation) in T4-2 cells, RT4-2 cells and S1 cells reseeded from collagen I to Matrigel for 12 hours (to only establish basal polarity).

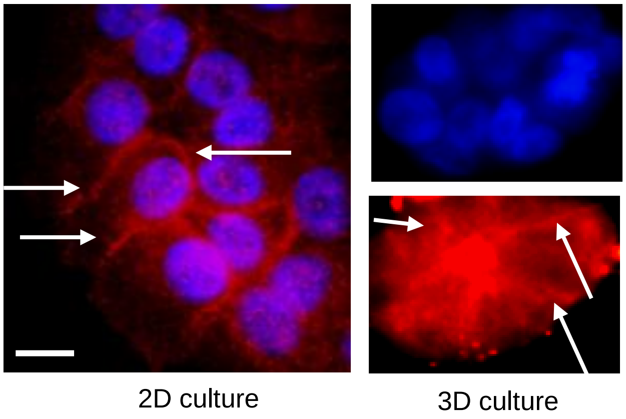

Fig. S2. Presence of NKCC1 in breast epithelial S1 cells. Immunostaining for NKCC1 (red) in S1 cells in 2D culture (on plastic) and 3D culture. Arrows indicate cell-cell location of NKCC1. The central accumulation of fluorescence in the glandular structure might be due to nonspecific high background in 3D culture. Nuclei are stained with DAPI (blue). Size bar, 5 µm

Journal of Cell Science • Supplementary information

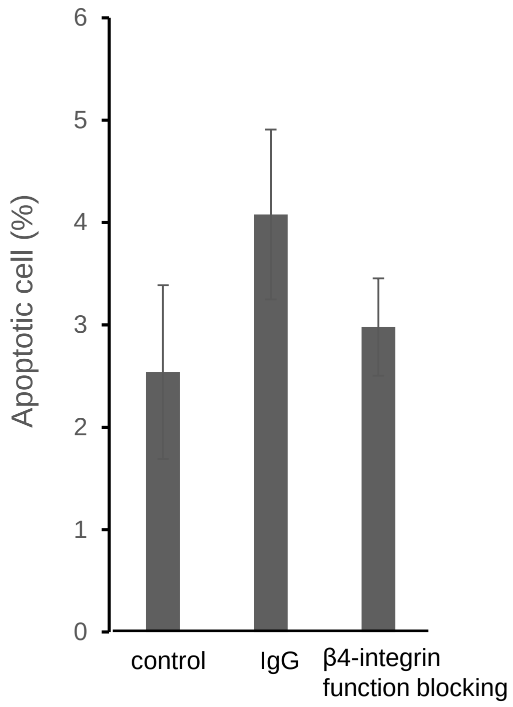

Fig. S3. Cell survival after β4-integrin blocking antibody treatment to prevent the establishment of basal polarity. Cells were cultured with the Matrigel drip method for 10 days, then released with dispase and incubated with 20 µg/ml IgG or β4-integrin blocking antibody for 30 minutes. Then, the multicellular structures were reseeded in the presence of Matrigel drip and cultured for seven days with 2.5 µg/ml IgG or function blocking antiβ4-integrin. In the 'control' group glandular structures were not released from Matrigel and were cultured for 17 days. Shown is the graph of the percentages of cells with apoptotic nuclei (e.g., smaller and dense; fragmented) based on DAPI staining. More than 200 nuclei were analyzed per condition in five biological replicates. No significant difference was detected.

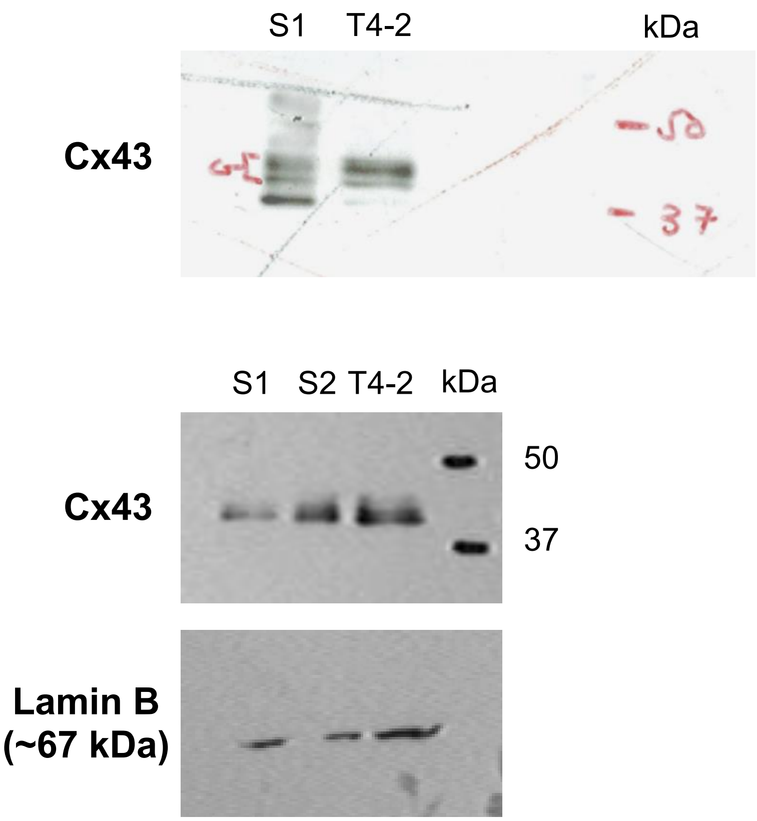

Fig. S4. Additional examples of western blots for Cx43 in 3D cultures of S1 and T4-2 cells. Note that on one of the blots, S2 cells were also used (they represent a ductal carcinoma *in situ* form in the HMT-3522 progression series). These examples combined with the one shown on the main figure 4A, illustrate the variety of Cx43 band patterns linked to heterogeneous mixtures of isoforms. Another example of blot with the internal control lamin B is also included.

Table S1. Original data for results presented. Tab 1 has data for Fig. 1A, tab 2 has data for Fig. 1B, tab 3 has data for Fig 2A, tab 4 has data for Fig 3B, tab 5 has data for Fig. 4B, tab 6 has data for Fig. 4C, and tab 7 has data for Fig 5B.

Available for download at
https://journals.biologists.com/jcs/article-lookup/doi/10.1242/jcs.260924#supplementary-data
